# Multidimensional metrics of niche space for use with diverse analytical techniques

**DOI:** 10.1038/srep41599

**Published:** 2017-02-01

**Authors:** Rachel E. Bowes, James H. Thorp, Daniel C. Reuman

**Affiliations:** 1 Kansas Biological Survey and Department of Ecology and Evolutionary Biology, University of Kansas, 2101 Constant Avenue, Lawrence, Kansas 66047-3759, USA; 2 Laboratory of Populations, Rockefeller University, 1230 York Ave., New York, NY, 10065, USA

## Abstract

Multidimensional data are integral to many community-ecological studies and come in various forms, such as stable isotopes, compound specific analyses (e.g., amino acids and fatty acids), and both biodiversity and life history traits. Scientists employing such data often lack standardized metrics to evaluate communities in niche space where more than 2 dimensions are involved. To alleviate this problem, we developed a graphing and analytical approach for use with more than two variables, based on previously established stable isotope bi-plot metrics. We introduce here our community metrics as R scripts. By extending the original metrics to multiple dimensions, we created n-dimensional plots and metrics to characterize any set of quantitative measurements of a community. We demonstrate the utility of these metrics using stable isotope data; however, the approaches are applicable to many types of data. The resulting metrics provide more and better information compared to traditional analytic frameworks. The approach can be applied in many branches of community ecology, and it offers accessible metrics to quantitatively analyze the structure of communities across ecosystems and through time.

The niche concept is a central organizing aspect of modern ecology and has been defined in many ways throughout the field’s history^1^,^2^,^3^,^4^,^5^,^6^. Although the niche concept is most prevalent in community ecology, it is used by ecologists working at many levels of ecological organization. For example, correlative niche models can be employed to estimate the spatio-temporal niches linking species occurrence to spatial datasets^7^, and mechanistic niche models are used in different theoretical frameworks depending on study aims^7^. One of the most prevalent niche concepts is that of Hutchinson’s n-dimensional hypervolume^1^,^8^. Recently, the concept of the ‘isotopic niche’ has gained popularity, using stable isotopic ratios to quantify a niche in isotope space^3^,^9^,^10^.

Many ecologists employ stable isotopes in food-web studies, using nitrogen (N), carbon (C), sulfur (S), oxygen (O), and/or hydrogen (H). δ^15^N (ratio of nitrogen isotopes, ^15^N to ^14^N, expressed relative to a standard) enriches stepwise with trophic transfers of biomass and is a major tool for estimating trophic position of organisms^11^,^12^,^13^. δ^13^C (ratio of carbon isotopes, ^13^C to ^12^C, expressed relative to a standard) can be used to determine original sources of dietary carbon, because it varies substantially among primary producers with different photosynthetic pathways (e.g. C_3_ vs. C_4_ photosynthetic pathways) and changes little with progression through a food web^11^,^13^,^14^,^15^. Estimates of the proportional contributions of food sources to the isotopic composition of the consumer’s tissues, which in turn reflects the assimilated diet of that consumer, can be achieved using mathematical mixing models^16^.

One of the most commonly used isotopic niche methods was developed by Layman *et al*.^17^,^18^. It has proven useful in measuring dispersion of carbon (δ^13^C) and nitrogen (δ^15^N) isotope ratios in bivariate space (i.e.ref. 18). Metric values first offered by Layman *et al*.^17^ included: (1) ranges for each variable considered (e.g., individual ranges of δ^13^C and/or δ^15^N); (2) mean distance to the centroid of points in 2-dimensional space (CD), which acts as a measure of species spread; (3) mean nearest neighbor distance in 2-dimensional space (NDD), functioning as a measure of density of species packing; (4) standard deviation of nearest neighbor distance (SDNND), measuring evenness of species packing in 2-dimensional space; and (5) total convex hull area, acting as a measure of the total amount of niche space occupied by the community. The graphical representation of communities and these measurements give researchers insight on trophic and/or isotopic diversity, the width of isotopic niche, the species isotopic redundancy, in addition to the overall distribution of resource use of a community.

The scientific applications of stable isotope methods have expanded tremendously in this century. For example, stable isotope signatures can be used to find patterns and mechanisms at the single organism level, assess the structure and dynamics of food webs, and trace the origins and migrations of species across the globe^19^,^20^. Isotopes have also been employed to follow whole ecosystem nutrient cycling in both terrestrial and marine systems and to examine global element cycles, past climatic conditions, hydrothermal vent systems, and rock sources^21^,^22^,^23^. As a consequence, isotopic analysis has become a standard instrument in the toolbox of many physiologists, ecologists, geochemists, and scientists studying element or material cycling in the environment.

Similar to carbon, the ratio of sulfur isotopes (δ^34^S) varies markedly among primary producers but changes relatively little with trophic transfers; it can also be used to identify important basal resources, especially in marine systems^24^,^25^,^26^. The δ^18^O and δ^2^H values of groundwater and precipitation vary at multiple spatial scales, allowing researchers to decipher patterns across small-scale environmental gradients^27^,^28^,^29^,^30^ or decode large-scale dietary patterns across geographic regions^31^,^32^,^33^. Traditionally, these elemental tracers are most frequently plotted and analyzed in a bivariate approach. Here, we offer meaningful metrics that can accommodate more axes and allow researchers to explore a greater number of niche dimensions simultaneously.

Ecologists now have the ability to detect the isotopic signatures of individual compounds occurring within bulk tissue, such as fatty acids, amino acids, and biomarkers^34^,^35^,^36^. Fatty acids represent a diverse group of molecules that comprise the majority of lipids in all organisms. Because of their biochemical restrictions and unique origins in plants and animals in some cases, fatty acids have proven to be a powerful tool in delineating food webs and assessing predator diets (e.g. ref. 37, 38, 39, 40, 41). Similar to fatty acids, amino acids are biologically important compounds; they are the dominant nitrogen-bearing biomolecules of organisms and are the structural monomers that comprise proteins. Patterns of isotopic fractionation during synthesis and transamination of amino acids can be used to determine trophic linkages, follow nutrient pathways, and distinguish between primary production sources (e.g. ref. 42, 43, 44, 45, 46, 47). Biomarkers are compounds that are produced by only a limited group of organisms. The use of biomarkers in ecological studies has risen in recent years, particularly in the field of microbial ecology. In the latter case, investigators have employed a combination of deliberately added tracers and isotopic analysis of biomarkers to directly link microbial identity (as assayed with the biomarker), biomass (the concentration of the biomarker in the habitat medium), and activity (isotope assimilation)^48^,^49^.

All these approaches have led to accumulation of multidimensional data and a better understanding of processes. Traditionally, these elemental tracers are most frequently plotted and analyzed in a bivariate approach, like the methods developed by Layman *et al*.^17^,^18^. Many studies have begun adding a third isotope into analyses to distinguish between similar systems^50^,^51^, and many isotope mixing models can be expanded to n-dimensions^16^. Properly assessing how and when extra dimensions add information to a problem, specifically by way of including additional isotope systems, is an intriguing and timely question. Here, we offer meaningful metrics that can accommodate more axes and allow researchers to explore a greater number of niche dimensions simultaneously as well as determine the extent to which these new axes are useful.

To enhance the ability of researchers to easily analyze data in three or more dimensions, we introduce here community metrics as R scripts. By extending the original Layman *et al*.^17^ metrics to multiple dimensions, we create n-dimensional plots and metrics useful for quantitatively characterizing a set of community measurements. The extensions and adaptations of these methods are elementary from a mathematical perspective, and thus should be easily employed by, and useful to most ecologists. We demonstrate the utility and ecological meaning of these newly-developed multidimensional metrics using stable isotopes; however, the approach is widely applicable to many other types of continuous, quantitative data.

## Results

Although our metrics can be applied to measure many kinds of community attributes (e.g., fatty acids, amino acids, traits), we demonstrate here the benefits as applied to stable isotope data. We use stable isotope data because: (a) this method is employed widely to assess the structure and dynamics of food webs; (b) such analyses often use two-dimensional Layman metrics; and (c) we believe that many of these bulk-tissue and amino acid isotope analyses as well as fatty-acid studies would benefit from multivariate analyses of three or more dimensions.

Numerous hypothesis-testing frameworks and analytical approaches have been proposed to characterize dispersion of carbon (δ^13^C) and nitrogen (δ^15^N) isotope ratios in bivariate space; however, the need for analytical tools in more dimensions is becoming increasingly evident. To illustrate the need for, and value of multidimensional metrics, we illustrate this below for various conceptual, ecological situations and by using a long-term museum data set.

**Figure 1:**
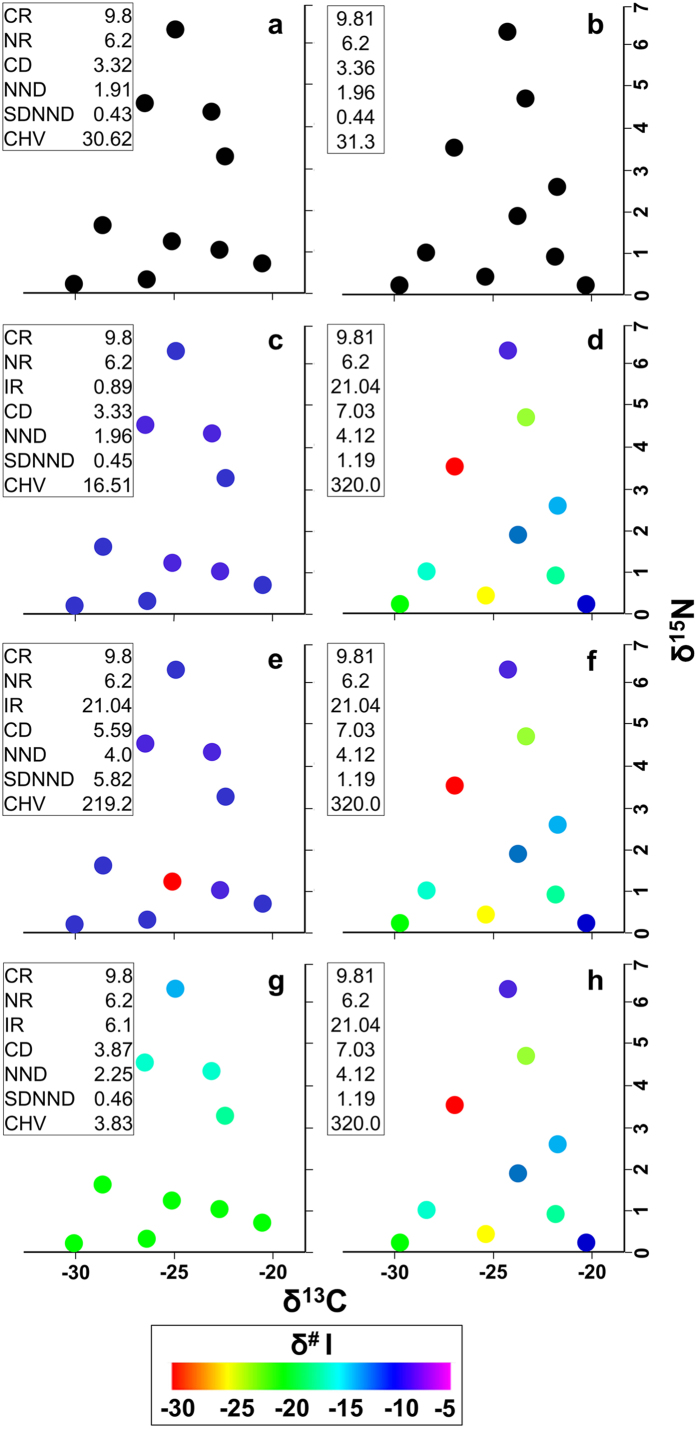
Schematic stable isotope plots (δ^15^N and δ^13^C) of two communities (community one on the left, community two on the right) in two (**a,b**) and three (**c–h**) dimensions. The first two dimensions are on the horizontal and vertical axes, and are the same in all plots for each community and virtually the same across communities. The third dimension is represented on c-h by colors corresponding to isotopic values ranging from −30 to −5, and show a variety of ways in which the communities may be revealed to differ when additional isotope data are collected. The third dimension represents a ratio for an arbitrary isotope, I, and is denoted δ^#^I. Each point represents a species. Euclidean community metric values are included for comparison: CR, δ^13^C range; NR, δ^15^N range; IR, δ^#^I range; CD, mean distance to the centroid; NND, mean nearest neighbor distance; SDNND, standard deviation of nearest neighbor distance; CHV, total convex hull area (2 dimensions) or volume (more than 2 dimensions). (**a,b**) The two theoretical communities look extremely similar in two dimensions, when only using δ^15^N and δ^13^C, with each of the metric values confirming their similarity. (**c–h**) When more information is added, the two communities differ (see text for details).

The first conceptual schematic (Fig. 1) shows how the metrics of two communities that are nearly identical in two dimensions could be influenced by a third dimension. The two theoretical communities look similar in two dimensions (Fig. 1a,b; when only using δ^15^N and δ^13^C), and all metric values are nearly identical. When more information is added as a third dimension (Fig. 1c–h; for example using another isotope, δ^#^I), it may be revealed in a variety of possible ways that the communities actually differ. The additional dimension could vary little (Fig. 1c) or widely (Fig. 1d), as revealed principally by the metric IR (δ^#^I range), but also reflected by other metrics. Alternatively, all taxa could be similar to each other except for one taxon (Fig. 1e), or all could vary widely (Fig. 1f), as reflected not by IR but by other metrics, especially SDNND. The additional dimension could be highly correlated with one of the previous dimensions (Fig. 1g) or may be uncorrelated (Fig. 1h). In the correlated case, the new dimension provides little additional information, and this is appropriately reflected in metric values that are mostly similar to the 2-dimensional measures (Fig. 1a). Only CHV has changed much, because it changes from a 2-dimensional area (Fig. 1a) to a 3-dimensional volume (Fig. 1g).

**Figure 2:**
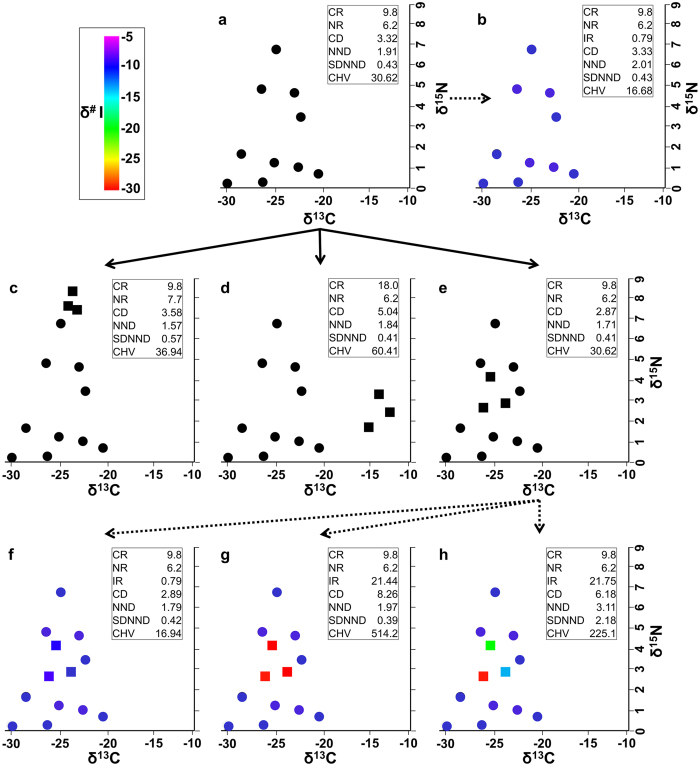
Schematic stable isotope bi-plots (δ^15^N and δ^13^C) of conceptual ways three new species might affect the metric values of a community in two (**a,c–e**) and three (**b,f–h**) data dimensions, with the third dimension being another possible isotope, δ^#^I, represented by colors. Points represent taxa, with the prior community members as circles and the new taxa by squares. Euclidean community metric value names are as in Fig. 1. (**a,b**) The original community in two (**a**) and three (**b**) dimensions. In this example, all species are similar with respect to their third-dimension (δ^#^I) values, and IR is very small. (**c–e**) Three examples of how three new species that are introduced into an existing community could alter community metrics in two dimensions. (**f–h**) Three examples of how assessment by three dimensions of data could reveal that the new species have different or additional impacts compared to assessment with two dimensions.

The second conceptual schematic demonstrates how the metrics of a community could be affected by the addition of three new species in two versus three data dimensions. Figure 2 presents a community of organisms prior to the addition of any new species in two (Fig. 2a) and three (Fig. 2b) dimensions. In the 3D example, all species are assumed similar with respect to the third dimension (δ^#^I) values, so IR is small (Fig. 2b). If three new species are introduced into the existing community and only two dimensions of isotopes are measured (Fig. 2c–e), the new species could, for example, differ from the original community in either the vertical dimension (Fig. 2c) or the horizontal dimension (Fig. 2d). The new species would then be reflected strongly in changes in the two dimensional metric values (Fig. 2c,d). However, if the new species did not differ markedly in the two measured dimensions (Fig. 2e), changes to the community would be difficult to detect using two dimensional metrics. The new species might, however, differ from the rest of the community when a third isotope ratio is measured (Fig. 2g,h). The new species could be similar to each other and differ from the existing community with regard to the third dimension (Fig. 2g), causing increases in IR, CD and CHV. Or the new species could differ not only from the existing community, but also from each other with respect to the third dimension (Fig. 2h), causing increases in IR, CD, NND, CDNND, and CHV (Fig. 2b–h). CD and CHV do not increase as dramatically from b to h as from b to g. The new species could, alternatively, be similar to the previous community with respect to the third dimension (Fig. 2f; δ^#^I) as well as the first two dimensions, showing little change in 3D as well as 2D metric values before and after invasion. Additional isotope dimensions beyond the third may yet reveal community effects of the added species. More than three isotope dimensions obviously complicate plotting, but the metrics for which we provide R code are easy to apply mathematically for any number of dimensions.

**Figure 3:**
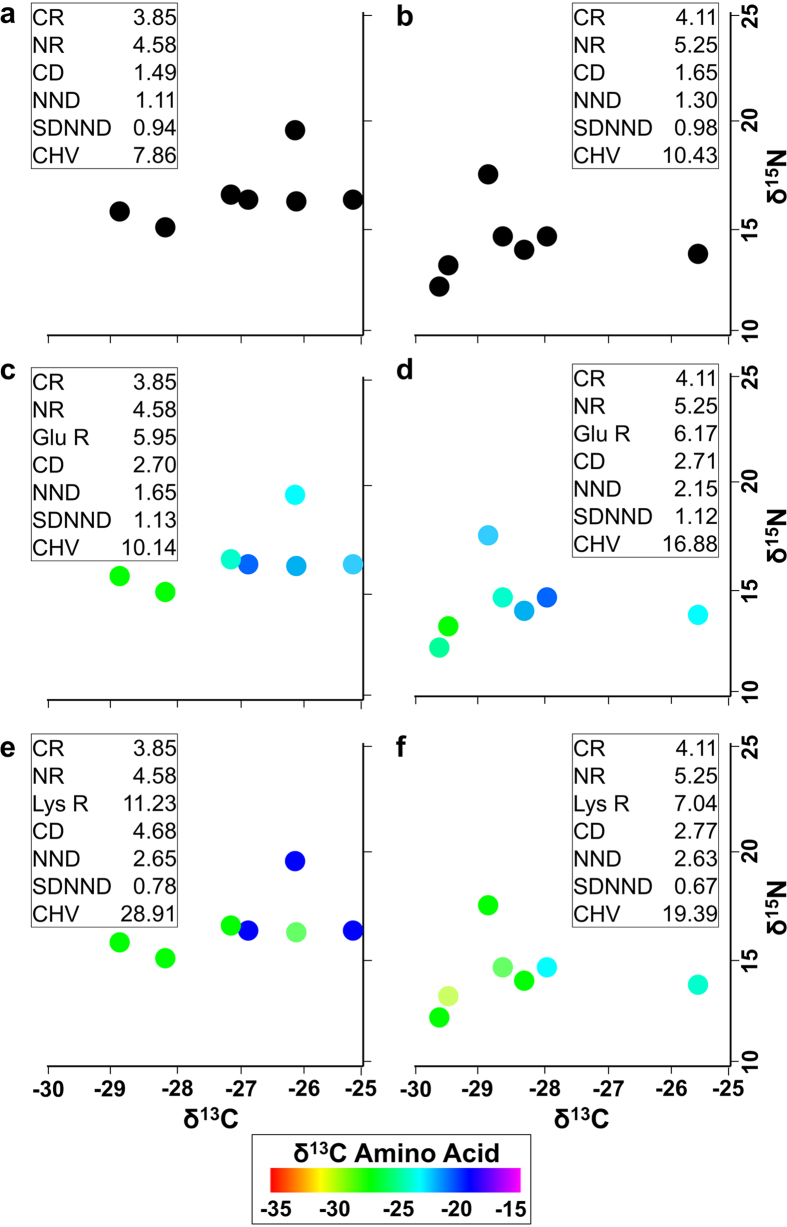
(**a,b**) Stable isotope bi-plots (δ^15^N and δ^13^C) of a community of fish in the Lower Ohio River before and after major dam construction. Each point represents a species’ mean value. Euclidean community metric values are included for comparison, metric names as in Fig. 1. In two dimensions the community looks very similar before (**a**) and after (**b**) dams were built. (**c,d**) Addition of a new dimension based on the δ^13^C signature for the amino acid Glutamic Acid before (**c**) and after (**d**) dam construction. (**e,f**) Addition of a new dimension for the δ^13^C signature for the amino acid Lysine before (**e**) and after (**f**) dam construction.

Finally, we demonstrate the utility of the metrics with real data from compound-specific stable isotope analysis (using δ^13^C and δ^15^N) of amino acids from fish collected before and after dam construction on the Lower Ohio River (Fig. 3). In two dimensions, the communities look similar before (a) and after (b) dam construction, and metric values are similar. The addition of a third dimension either alters apparent relationships among fish species or has little effect on our perception of the communities, depending on which amino acid is used. This may reflect the fact that some dimensions can contain more information about trophic diversity than others. Relationships among species seemed to change very little from before (Fig. 3c) to after (Fig. 3d) dam construction when we added the δ^13^C signature for Glutamic Acid (isotopic range from −35 to −15). In contrast, substantial differences were evident in community relationships from before (Fig. 3e) to after (Fig. 3f) dam construction when using the δ^13^C signature for Lysine, an essential amino acid. These differences were confirmed by the fact that resampling-based 95% confidence intervals for Lysine range (Lys R) and centroid distance (CD) before dam construction did not include the same metrics after dam construction (see Supplemental Materials for confidence intervals).

**Figure 4:**
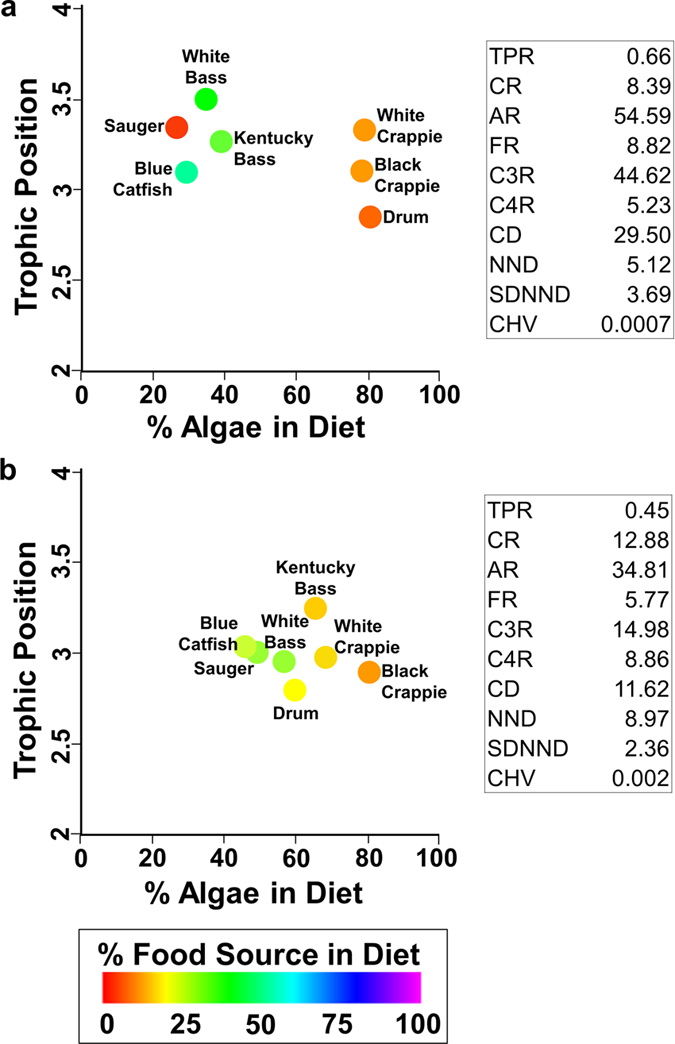
Fish in the Lower Ohio River before (**a**) and after (**b**) major dam construction. Plots consist of trophic position versus percent algae contribution to diet, evaluated by compound-specific stable isotope analysis of amino acids (δ^15^N and δ^13^C). Each point is a species’ mean value. Color shows percent C_3_ terrestrial plant contribution to the diet. Euclidean community metric values are included for trophic position range (TPR); % cyanobacteria range (CR); % algae range (AR); % fungi range (FR); % C_3_ terrestrial plants range (C3R); % C_4_ terrestrial plants range (C4R); and the metrics defined previously (see Fig. 1 for abbreviations).

We can delve deeper into what the new amino acid isotope values and metrics mean about community structure before and after dam construction by calculating fish trophic positions and the proportion of different food sources in fish diets (Fig. 4). Compound-specific carbon isotope values of amino acids and a Bayesian mixing model were used to compute the percentages of algae, C_3_ terrestrial plants, C_4_ terrestrial plants, aquatic macrophytes, and cyanobacteria in the fish species’ diets^52^,^53^. Trophic position was calculated with compound-specific nitrogen isotope values of Phenylalanine and Glutamic Acid using a trophic position equation^46^,^47^,^54^,^55^. Metric values were then calculated using all 6 of these new dimensions (though only three dimensions could be plotted at one time). Before dam construction (Fig. 4a), four species differed substantially from the other three. After dam construction (Fig. 4b), however, many of the ranges shifted and CD and SDNND decreased. Moreover, species-specific shifts in diet and trophic position were evident, e.g., all species clustered together after dam construction. Resampling based 95% confidence intervals for TPR, AR, FR, C3R, and CD before and after dam construction did not include the values of the same metrics computed based on data from after dam construction was significant due to non-overlapping 95% confidence intervals created using a resampling protocol (Methods, see Supplemental Materials for confidence intervals).

## Discussion

Higher-dimensional community metrics provide additional information and may reveal the influence of new dimensions of ecology on our conclusions about community structure, as compared to traditional analytical frameworks with fewer dimensions of data. Higher metric values indicate more spread in the overall community structure in niche space with respect to the available measurements for assessing niche space, with each metric value reflecting a different measure of spread in that space. According to Layman *et al*.^17^, CD shows species spread in space, NND indicates density of species packing or niche redundancy, SDNND corresponds with the evenness of species packing in niche space, and CHV reflects the total amount of niche space occupied. As seen in Figs 1–4, the addition of a third and higher dimensions of data has the potential to reveal, through plots and our generalized metrics, a great deal of additional information pertinent to community structure. Third and higher dimensions of data can confirm (Fig. 1c) or strongly modify (Fig. 1d,f,h) conclusions based only on two dimensions.

Our figures demonstrate the benefits of analyzing communities in more than two dimensions. Many distinguishing characteristics between communities are not readily visible when only two niche dimensions are used, in particular when trying to decipher feeding relations with isotopes, as the examples used here suggest. Although these demonstrations are in two and three dimensions, and the differences in community structure are easily distinguished using the plots alone, it is possible to calculate the metrics with any number of dimensions (e.g., compound specific analyses) and in that context differences in community structure could be gleaned from metrics but not from bi- or tri-plots in an efficient manner.

Our example with real data not only illustrates how the addition of another dimension can potentially show differences between two communities (Fig. 3), but also resolves some of the limitations identified^56^ with the metrics originally presented by Layman *et al*.^17^. One of the biggest issues with Layman metrics is how sensitive they are to sample sizes. Our resampling procedure takes into account sample size when creating confidence intervals, so sample sizes are automatically accounted for (Methods).

Another limitation of the original metrics^17^ is that the observed patterns could be a function of baseline variability and not reflect true differences among consumers, and metric values could be misleading or deceptive when basal source bulk signatures (δ^13^C) overlap. Rather than inferring food source use from relative spacing of consumers in δ^13^C-δ^15^N bi-plots, an ecologist can now use baseline-corrected trophic position estimates instead of absolute δ^15^N values in bivariate plots (with bulk-tissue signatures:^57^,^58^; with compound specific isotope analysis of amino acids:^46^,^47^,^55^) and quantify the relative dietary importance of basal food sources using mixing models (e.g., FRUITS model^52^,^53^), converting δ-space to %-space (dietary percent of basal sources; Fig. 4 and ref. 3). One can then use our community metrics to quantify a community and compare between communities.

There have been recent developments using multivariate ellipse-based metrics via a Bayesian approach to assess isotopic niches across communities^59^. In a Bayesian approach, Bayes’ theorem is used to update the probability for a hypothesis as more evidence or information becomes available. This means that there are post-hoc assumptions and uncertainty, which is very valuable when measuring dispersion relative to changes in sample size^59^. This is not always appropriate or necessary, however, especially when just assessing overall dispersion of a community. Here we offer metrics which are computationally very simple for use by researchers that do not wish to employ for various reasons a Bayesian approach.

Our freely available, community metrics in R scripts are easily adapted to a number of different data types. The metrics allow for the comparison of populations and communities, illuminating differences not readily apparent in one or two dimensions. The only requirements are that the number of species be greater than the number of variables or axes and that the calculations of metric values be based on the same variables. For instance, when dealing with fatty acid signatures, researchers often report differences in fatty acid content of different primary production sources and how those relate to the fatty acid content of a consumer’s tissues. With the metrics offered here, researchers can now quantitatively compare these differences across all species surveyed simultaneously, detecting overlap in feeding habits of consumers and spread within a community.

Moving beyond the qualitative descriptions of relative position in isotope-space (or fatty-acid space, etc.), our community metrics provide a means for basic comparisons among food webs or other community properties. Although the demonstrations here are with isotope data and food webs, the analysis could be adopted for other kinds of data. Our very simple generalization of the metrics of Layman *et al*.^17^, with the advent of new technologies and the increase in availability of multidimensional data, can provide insight into community structure with little difficulty.

## Methods

### Compound Specific Stable Isotope Analysis Methods

#### Museum fish samples for the Lower Ohio River

Museum collections and species surveys by government agencies provide data potentially useful for analyzing long-term environmental impacts^60^,^61^ as well as spatially dispersed ecological processes. We analyzed food sources and trophic position of piscivorous and invertivorous fishes from the Lower Ohio River (Evansville, Indiana to Cairo, Illinois USA) using preserved specimens from museums. Samples were donated by the Bell Museum, Field Museum, Illinois Natural History Survey, Illinois State Museum, Milwaukee Public Museum, Ohio State University Museum of Biological Diversity, Southern Illinois University, University of Michigan Museum of Zoology, and University of Wisconsin - Stevens Point. The largest preserved specimens were chosen for tissue harvesting; however, museum specimens of fish tend to be small in general, reflecting the need to conserve limited shelf space.

#### Sample processing and isotope analysis of fish tissue from the Lower Ohio River

We extracted muscle tissues from an area between the lateral line and dorsal fin of adult fish preserved in today’s museums in ethyl alcohol and probably previously for short or long periods in formalin. Neither preservative significantly alters the isotopic results^62^,^63^. Tissue samples were rinsed with deionized water, placed in pre-combusted glass vials, dried at 60 °C for 48 hr, and then ground into a fine, homogenized powder using a Wig-L-Bug mixer/amalgamator.

After samples were dried, powdered, and homogenized, their δ^13^C and δ^15^N bulk tissue and amino acid stable isotope ratios were determined at the UC-Davis Stable Isotope Facility. The data for each bulk tissue sample included total N and C and δ^13^C and δ^15^N values. The δ^13^C and δ^15^N values were determined based on the relative difference in isotopic ratio between the samples and known standards, as represented by the following equation: δX = ((R_sample_/R_standard_) − 1) × 1000 where X is ^13^C or ^15^N, and the corresponding ratio is R = ^13^C/^12^C or R = ^15^N/^14^N respectively. Vienna Pee Dee Belemnite is used as the standard ratio for carbon, and atmospheric nitrogen was used as the N standard. All isotope ratios are given in per mil (‰).

General techniques for compound specific isotope analysis of amino acids (AA-CSIA) are summarized below and extensively described in Walsh *et al*.^43^. Sample preparation involves acid hydrolysis for the liberation of amino acids from proteins and derivatization by methyl chloroformate to produce compounds amenable to gas chromatography (GC) analysis. Amino acid derivatives are injected in split (^13^C) or splitless (^15^N) mode and separated on an Agilent J&W factor FOUR VF-23 ms column (30 m × 0.25 mm ID, 0.25 micron film thickness). Once separated, amino acid derivatives are quantitatively converted to CO_2_ and NO_x_ in an oxidation reactor at 950 °C, and NO_x_ are subsequently reduced to N_2_ in a reduction reactor at 650 °C. Following water removal through a nafion dryer, N_2_ or CO_2_ enters the isotope-ratio mass spectrometry (IRMS). A pure reference gas (CO_2_ or N_2_) is used to calculate provisional δ-values of each sample peak. Next, isotopic values are adjusted to an internal standard (e.g. norleucine) of known isotopic composition. Final δ-values are obtained after adjusting the provisional values for changes in linearity and instrumental drift such that correct δ-values for laboratory standards are obtained. Signatures of both δ^13^C and δ^15^N were determined for the following amino acids and expressed as per mil (‰): Alanine, Aspartic Acid, Glutamic Acid, Glycine, Isoleucine, Lysine, Methionine, Phenylalanine, Proline, Tyrosine, and Valine. Tyrosine signatures were excluded from analyses dues to missing measurements caused by concentrations below detection limits.

#### Trophic position and food source calculations using amino acids

To calculate trophic position of consumers from AA-CSIA data, we employed the following formula: TP = [((δ^15^N of Glutamic Acid − δ^15^N of Phenylalanine) − 3.4) ÷ 7.6] + 1 (e.g., ref. 46, 47, 54, 55, 62, 64 and 65).

To calculate the amino acid composition of food sources, we measured isotopic signatures using δ^13^C AA-CSIA for three replicates of the following potential aquatic and terrestrial food sources, as represented biochemically by cyanobacteria (*Spirulina*), green algae (*Chlorella sp.*), fungi (baker’s yeast or *Saccharomyces cerevisiae*), a C_4_ terrestrial plant (corn, *Zea mays*), and the following C_3_ plants: the grass *Elymus sp*. (probably *E. virginicus*), cottonwood tree leaves (*Populus deltoides*), soybeans (*Glycine max),* and an aquatic vascular macrophyte (wild celery, *Valisneria americana*). These specific food sources were chosen as they represent common food sources available in rivers across the USA. The terrestrial sources were collected in Lawrence, Kansas, and aquatic sources were ordered from laboratory cultures (PureBulk.com). These new signatures were used in conjunction with data from other aquatic studies^45^,^66^ to determine classification and specific isotopic fingerprints of the different food sources.

δ^13^C values of each of the amino acids were normalized to their respective sample means (δ^13^C_AA_ − mean δ^13^C_AA_) and tested for univariate normality. Normalizing the values to the means removes any effect of growth media between the different food sources. To explore patterns and determine producer food groups we performed principal component analysis on normalized δ^13^C signatures of all available amino acids. This analysis showed that samples clustered according to major phylogenetic associations (5 major groups were identified: cyanobacteria, algae, fungi, C_3_ plants, and C_4_ terrestrial). Amino acid δ^13^C signatures between these different producer groups were tested with ANOVA. We then performed linear discriminant function analysis on δ^13^C AA-CSIA to determine the combination of δ^13^C AA-CSIA values (independent variables, in this case 9 amino acids: Alanine, Aspartic Acid, Glutamic Acid, Glycine, Isoleucine, Lysine, Phenylalanine, Proline, and Valine) that best explained differences between food sources (categorical variables determined by principal component analysis), and we used a leave-one-out cross validation approach to calculate the probability of food source group membership of the classifier samples. To test that there were no difference in classification between the groups, Pillai-Bartlett trace (MANOVA) was applied. All preliminary analyses on food sources were done in Minitab 14 (Minitab Inc., State College, PA, USA).

Relative contributions of dietary amino acids to consumers were estimated using the software “Food Reconstruction Using Isotopic Transferred Signals” (or FRUITS;^52^,^53^). Normalized δ^13^C values as well as their associated uncertainties (±1 S.D.), for each consumer species and potential food sources in the river were inputs into the FRUITS model. FRUITS incorporates the capability to account for dietary routing; that is, the contribution of different original primary production sources towards the amino acids signals measured in the consumer. It was assumed that all food sources were equally likely and had the potential to make up 100% of the diet of the consumer. No other priors were used in the model. FRUITS is executed with a software package for performing Bayesian inference Using Gibbs Sampling (BUGS), and also considers the biochemical composition of sources and which sources are most likely to contribute the most (see http://www.mrc-bsu.cam.ac.uk/software/bugs/). This tool is also sensitive to trophic fractionation. The FRUITS output is a summary of percent contributions of each potential food source to the consumer’s diet along with standard deviation and confidence intervals. FRUITS version 2.0 (http://sourceforge.net/projects/fruits/) was used for estimating food source contributions. Taking into account posterior uncertainties in the proportional contributions of different food sources and food source combinations, sensitivity analyses were conducted to evaluate the reliability of the results^52^.

### Metrics

All computations of metrics were done in R version 3.2.1 with the included code (Supplemental Materials). Here and henceforth, 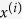 is the *n*-vector of isotope or other community data for species 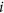, *S* is the number of taxa or species for which data are available, and


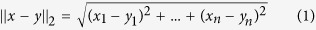


is the Euclidean distance (Equation 1),

Range (R): Total distance between the farthest-separated taxa with respect to that axis (i.e. maximum minus minimum). Mean distance to the centroid (CD): Average Euclidean distance of each species to the centroid, where the centroid is the mean value of each axis for all species.

According to Layman *et al*.^17^, this metric functions as a measure of species spread. Mean nearest neighbor distance (NND): Mean of the Euclidean distances to each species’ nearest neighbor in *n-*dimensional space. According to Layman *et al*.^17^, this metric functions as a measure of density of species packing. Standard deviation to the nearest neighbor (SDNND): The standard deviation of the nearest neighbor distances in *n*-dimensional space. According to Layman *et al*.^17^, this metric acts as a measure of evenness of species packing in n-dimensional space. Convex hull volume (CHV): Convex hull area (2 dimensions) or volume (more than 2 dimensions), is the volume encompassed by all species in the *n*-dimensional space. According to Layman *et al*.^17^, this metric is a measure of the total amount of niche space occupied by the community. Our code simply calls the convhulln function in the geometry package of R, which in turn calls the Qhull library (www.qhull.org).

#### Resampling and confidence intervals

When individual-level measurements were available, i.e., several individuals of each taxon were captured and isotope measurements were made for each individual, confidence intervals for metric values were constructed through a non-parametric resampling scheme: individuals for each taxon were selected with replacement, species mean isotope values were recomputed, and metrics were recalculated 10,000 times to get resampling distributions of all metric values. For instance, if *k* individuals of a taxon were captured and measured in the original dataset, *k* individuals were selected, with replacement, from this set to help form the surrogate datasets used in the scheme. Resampling was independent for different species. This kind of resampling is a standard approach.

Individual data were not available for the results of Fig. 4, because taxon-mean isotope values were processed through the Bayesian mixing model software FRUITS to get the trophic position and percent-diet estimates used in that figure. However, FRUITS provides not only point estimates of output information, but also standard error estimates. Therefore, to get confidence intervals for our metrics, we carried out a parametric resampling scheme where values were assumed to come from normal distributions with the mean and standard deviations given by FRUITS, though truncated to mathematically possible ranges (e.g., diet fraction values are between 0 and 1, inclusive). Resampling was done independently across species and measurements. This is another standard resampling approach.

## Additional Information

**How to cite this article**: Bowes, R. E. *et al*. Multidimensional metrics of niche space for use with diverse analytical techniques. *Sci. Rep.* **7**, 41599; doi: 10.1038/srep41599 (2017).

**Publisher's note:** Springer Nature remains neutral with regard to jurisdictional claims in published maps and institutional affiliations.

## Supplementary Material

Supplementary Material

Supplementary Information
