# Supplementary material for: Multidimensional metrics of niche space for use with diverse analytical techniques
Source: Sci Rep. 2017 Feb 1;7:41599. doi: 10.1038/srep41599 (PMC5286414; doi:10.1038/srep41599)
Supplement: Supplementary Material [file srep41599-s1.pdf]

Supplemental Material: Multidimensional metrics of niche space for use with diverse analytical techniques

Rachel E. Bowes[1], James H. Thorp[1*], and Daniel C. Reuman[1,2]

[1]Kansas Biological Survey and Department of Ecology and Evolutionary Biology, University of Kansas, 2101 Constant Avenue, Lawrence, Kansas 66047-3759, USA

[2]Laboratory of Populations, Rockefeller University, 1230 York Ave., New York, NY, 10065, USA

*Email: thorp@ku.edu

Confidence intervals for Figures 3 & 4

All 95% confidence intervals, obtained by taking the 2.5% and 97.5% quantiles on resampling, for metric values in Figure 3.

Figure 3 A & B.

	**Before Dam**			**After Dam**		
**Metric**	**Low 95% CI**	**Metric Value**	**High 95% CI**	**Low 95% CI**	**Metric Value**	**High 95% CI**
**CR**	2.60	3.85	5.77	3.46	4.11	6.71
**NR**	3.20	4.58	6.70	2.37	5.25	9.20
**CD**	1.34	1.49	2.11	1.29	1.65	2.65
**NND**	0.86	1.11	1.78	0.94	1.30	2.22
**SDNND**	0.39	0.94	1.34	0.50	0.98	2.05
**CHV**	5.22	7.86	16.46	5.15	10.43	24.55

Figure 3 C & D.

	**Before Dam**			**After Dam**		
**Metric**	**Low 95% CI**	**Metric Value**	**High 95% CI**	**Low 95% CI**	**Metric Value**	**High 95% CI**
**CR**	2.60	3.68	5.77	3.46	4.11	6.71
**NR**	3.20	4.58	6.70	2.37	5.25	9.20
**Glu R**	4.62	5.95	9.53	4.65	6.17	8.88
**CD**	2.29	2.70	3.46	2.29	2.71	3.80
**NND**	1.46	1.65	2.77	1.62	2.15	3.26
**SDNND**	0.28	1.13	1.59	0.52	1.12	1.98
**CHV**	6.45	10.14	22.11	7.90	16.88	44.67

Figure 3 E & F.

	**Before Dam**			**After Dam**		
**Metric**	**Low 95% CI**	**Metric Value**	**High 95% CI**	**Low 95% CI**	**Metric Value**	**High 95% CI**
**CR**	2.60	3.68	5.78	3.46	4.11	6.71
**NR**	3.18	4.58	6.70	2.37	5.25	9.20
**Lys R**	8.74	11.23	15.67	4.53	7.04	19.47
**CD**	3.74	4.68	5.99	2.40	2.77	5.44
**NND**	2.14	2.65	3.64	1.90	2.63	4.32
**SDNND**	0.44	0.78	1.91	0.50	0.67	3.52
**CHV**	15.33	28.91	64.42	9.54	19.39	85.26

All 95% confidence intervals, obtained by taking the 2.5% and 97.5% quantiles on resampling, for metric values in Figure 4.

Figure 4 A & B.

	**Before Dam**			**After Dam**		
**Metric**	**Low 95% CI**	**Metric Value**	**High 95% CI**	**Low 95% CI**	**Metric Value**	**High 95% CI**
TPR	0.46	0.66	1.08	0.34	0.45	1.41
CR	7.96	8.39	31.73	8.95	12.88	43.08
AR	48.13	54.59	88.75	24.78	34.81	79.83
FR	6.87	8.82	21.78	5.61	5.77	21.31
C3R	37.36	44.62	82.67	13.71	14.98	68.32
C4R	4.12	5.23	19.07	7.01	8.86	22.87
CD	26.08	29.50	39.79	10.37	11.62	29.83
NND	4.85	5.12	24.43	8.01	8.97	25.91
SDNND	2.24	3.69	13.44	2.15	2.36	15.78
CHV	0	0.0007	243.96	0	0.002	305.84
